# A New Paradigm for KIM-PTP Drug Discovery: Identification of Allosteric Sites with Potential for Selective Inhibition Using Virtual Screening and LEI Analysis

**DOI:** 10.3390/ijms222212206

**Published:** 2021-11-11

**Authors:** James Adams, Benjamin P. Thornton, Lydia Tabernero

**Affiliations:** 1 School of Biological Sciences, Faculty of Biology Medicine and Health, Manchester Academic Health Science Centre, University of Manchester, Manchester M13 9PT, UK; jadams_91@hotmail.co.uk (J.A.); benjamin.thornton-3@postgrad.manchester.ac.uk (B.P.T.); 2 Lydia Becker Institute for Immunology and Inflammation, University of Manchester, Manchester M13 9PT, UK; 3 Antimicrobial Resistance Network, University of Manchester, Manchester M13 9PT, UK

**Keywords:** protein phosphatases (PPases), phosphatase inhibitors, hematopoietic protein tyrosine phosphatase (HePTP), striatum-enriched protein tyrosine phosphatase (STEP), protein tyrosine phosphatase SL (PTP-SL), kinase interaction motif protein tyrosine phosphatases (KIM-PTPs), computational screening, virtual screening (VS), VSpipe, ligand efficiency indices (LEIs), drug discovery

## Abstract

The kinase interaction motif protein tyrosine phosphatases (KIM-PTPs), HePTP, PTPSL and STEP, are involved in the negative regulation of mitogen-activated protein kinase (MAPK) signalling pathways and are important therapeutic targets for a number of diseases. We have used VSpipe, a virtual screening pipeline, to identify a ligand cluster distribution that is unique to this subfamily of PTPs. Several clusters map onto KIM-PTP specific sequence motifs in contrast to the cluster distribution obtained for PTP1B, a classic PTP that mapped to general PTP motifs. Importantly, the ligand clusters coincide with previously reported functional and substrate binding sites in KIM-PTPs. Assessment of the KIM-PTP specific clusters, using ligand efficiency index (LEI) plots generated by the VSpipe, ascertained that the binders in these clusters reside in a more drug-like chemical–biological space than those at the active site. LEI analysis showed differences between clusters across all KIM-PTPs, highlighting a distinct and specific profile for each phosphatase. The most druggable cluster sites are unexplored allosteric functional sites unique to each target. Exploiting these sites may facilitate the delivery of inhibitors with improved drug-like properties, with selectivity amongst the KIM-PTPs and over other classical PTPs.

## 1. Introduction

Protein tyrosine phosphatases (PTPs) are fundamental regulators of numerous biological pathways and important therapeutic targets for multiple diseases [1,2,3,4,5,6]. A subfamily of PTPs are the kinase interaction motif protein tyrosine phosphatases (KIM-PTPs): HePTP, PTPSL and STEP. KIM-PTPs are involved in the negative regulation of mitogen-activated protein kinase (MAPK) signalling pathways [7,8,9,10], and represent important therapeutic targets for cancer [11,12], or neurodegenerative diseases [13,14].

Given their therapeutic importance, several investigations into their inhibition have also been undertaken [15,16,17,18], together with their activation [19]. However, inhibition of PTPs has historically been problematic because of the highly conserved active site and its polar nature [20]. Focus has now turned to target regulatory mechanisms, including allosteric regulation, protein oligomerisation and redox modulation [21,22,23].

Particularly, targeting sites distal to the active site offers new opportunities to develop compounds with increased selectivity and drug-like properties [23]. Examples include the targeting of different conformational states of RPTPG [24], allosteric sites of PTP1B [25,26] and SHP2 [27], or the trimer interface of PRL [28].

The development of allosteric inhibitors raises a new challenge in the identification of the allosteric site for subsequent targeting often requiring different biochemical and structural methods, which are time consuming. Computational approaches to find ligand binding sites are widely used because they are fast, cheaper and a good complement to experimental methods. We recently reported the use of the virtual screening tool, VSpipe [29], to effectively identify functional ligand binding sites on PTP1B, including an allosteric inhibitor site, and on fungal phosphatases [30]. The ability of VSpipe to perform rapid blind docking of compound libraries, together with the assessment of the ligand efficiency indices (LEIs) of the ligands [31,32], facilitates a comparison of the chemical–biological space of binders at different regions, thus guiding the selection of the most druggable sites for compound development.

The aim of this study was two-fold; first, to identify novel and druggable ligand binding sites in KIM-PTPs that define a more favourable chemical–biological space than the highly polar active site. Second, to explore if those druggable sites may offer selective inhibition across different families of PTPs. For this we have used VSpipe, a virtual screening pipeline, on the three KIM-PTPs and defined a ligand cluster distribution that is unique to this subfamily of PTPs, with several clusters located on KIM-PTP sequence specific motifs [33]. This is in contrast to the cluster distribution obtained for PTP1B [29] that mapped mainly to the conserved classic PTP motifs [34].

Assessment of the unique KIM-PTP binding clusters using LEI plots ascertained that the binders at the KIM-PTP specific clusters reside in a more drug like chemical–biological space than that of binders at the active site. Further to this, LEI analysis demonstrated that the chemical–biological space in which binders of KIM-PTP specific clusters reside is unique to each phosphatase. Structural analysis defined key features responsible for the differences at the cluster binding sites. These features may be used in the design and development of inhibitors that are selective amongst the KIM-PTPs and that have more drug-like properties.

## 2. Results and Discussion

### 2.1. VSpipe Blind Docking Comparison of the KIM-PTPs

Targeting of the open, super-open and allosteric sites has been suggested as an approach to yield compounds of improved physiochemical properties when compared to those of active site inhibitors [23]. Thus, the receptor models for the three KIM-PTPs used in this study were all in the open conformation and obtained from crystallographic structures deposited in the protein data bank for PTP-SL (PDB ID: 1JLN), STEP (PDB ID: 2BV5) and HePTP (PDB ID: 3O4U). However, in the open structure of HePTP, due to the flexibility of certain regions, there are missing residues in the deposited structures [35]. Therefore, we used Modeller [36] to generate a complete model (see methods for details). For the open form structure 3O4U, residue sequences ^257^DHQTP^261^ (downstream of the WPD-loop) and ^196^QLREGKEKC^204^ (E-loop) were modelled (Supplementary Figure S1). The ^257^DHQTP^261^ loop is very flexible, thus caution is advisable when considering the exact conformation of this region. However, confidence in the model generated can be drawn from similarities to previous examples in the literature; for example, the structure of the super-open form of RPTPγ that was exploited to obtain nanomolar range inhibitors [24].

To aid identification of new ligand binding sites on the KIM-PTPs, we carried out blind docking with VSpipe [29] against each KIM-PTP, using the Asinex fragment library (composed of 6243 chemical fragments).

**Figure 1:**
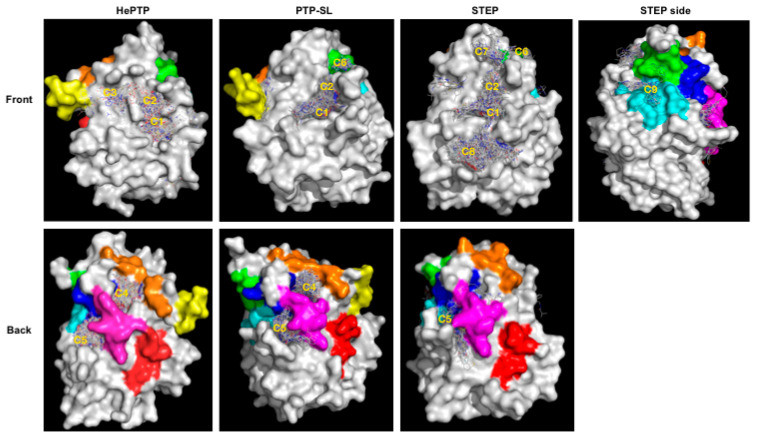
Blind docking results for kinase interaction motif protein tyrosine phosphatases KIM-PTPs (HePTP, PTPSL and STEP). The structure of the three KIM-PTPs are shown as a grey surface with key functional KIM-PTP specific motifs [33] coloured as follows: K2 in yellow, K3 in orange, K4 in red, K5 in magenta, K6 in cyan, K7 in blue, K8 in green. Compounds are shown as lines. Areas where there is a high density of compounds (greater than 10) are classified as clusters and numbered: C1, active site; C2, open form pocket; C3, substrate-binding site; C4, centred between K3, K5 and K7; and C5, centred on K5, K6 and K7; C6, centred on K8; C7 above open pocket; and C8 below the active site. The front view and back view for all three KIM-PTPs are shown. For STEP, a side view is also shown to see cluster C9 on K6.

Blind docking identified several ligand clusters (a cluster being greater than ten fragments at a specific site) at different binding sites (Figure 1). To aid our understanding of the functional relevance of these clusters, we mapped onto the structures the previously identified unique sub-family sequence motifs specific for KIM-PTPs (K2-K8), not shared by other PTPs [33] (Figure 1). Specifically, the location of the five clusters identified for HePTP were as follows: C1, active site; C2, open pocket; C3, a site of importance for p-ERK peptide binding [37]; C4, K3, K5 and K7 motifs; C5, K5 to K7 motifs. The C2 binding site is created by the open conformation of WPD loop that reveals a pocket above the active site.

For PTP-SL, blind docking identified five clusters of fragments (Figure 1). Four of them are located at the sites identified for HePTP: C1, active site; C2, open pocket; C4, K3, K5 and K7 motifs; and C5, K5 to K7 motifs. A further cluster, C6, that resides at the K8 motif, was also observed. Interestingly, C3 was not identified for PTP-SL, suggesting potential for selectivity. For STEP, seven clusters of fragments were identified (Figure 1), many located at sites that were common to HePTP and PTP-SL: C1, active site; C2, open pocket; C5, K5 to K7 motifs; and C6, K8 motif; whilst the C4 site found for HePTP and PTP-SL represented a low density binding site (defined as less than 10 fragments) in STEP. However, there were additional clusters in STEP: C7, in-between the open pocket and K8; C8, below the active site; and C9, a cluster that resides between K6 and K8 motifs. Clusters C7 and C8 were not identified on the other two KIM-PTPs, thus these sites may afford opportunities for selective interaction, although functional importance of these sites is unclear. C9 is more interesting as it only appears in STEP and also locates to the KIM-PTP specific motifs K6 and K8.

In summary, blind docking identified several ligand clusters, some of which reside at sites that are unique to the KIM-PTPs, exploring binding capabilities beyond the conserved active site. Differences are also apparent between the three KIM-PTPs, suggesting these different binding sites may offer opportunities to exploit selectivity.

### 2.2. Ligand Efficiency Analysis of Binders at Clusters 1, 4, 5 and 6

Clusters 4, 5 and 6 are located on KIM-PTP specific motifs and sites associated with clusters 4 and 5 were identified as potential druggable pockets (score > 0.5) by DoGSiteScorer (DGSS) [38] (Table 1). DGSS measures the volume of any pocket of interest and the amino acid composition, and calculates a druggability score, which indicates whether it is likely drug-like compounds will interact with the pocket. For STEP, DGSS identified two small pockets associated with C6 which we refer to as C6 and C6′, although the score is low. For HePTP, DGSS identified a large pocket including C1 and C2 (Table 1) with high druggability score. Thus, we decided to further explore these KIM-PTP specific clusters (C4–C6) by conducting targeted docking with VSpipe-Vina [29] at the associated pockets and at the active site (C1) for comparison.

Targeted docking at C1, C4, C5 and C6 was done with VSPipe using the Chembridge Diverset library (50,000 compounds) for all three KIM-PTPs with the same models as for the blind docking. The results of the docking were sorted by binding affinity (ΔG) and filtered to select the top 500 binders for further analysis.

**Figure 2:**
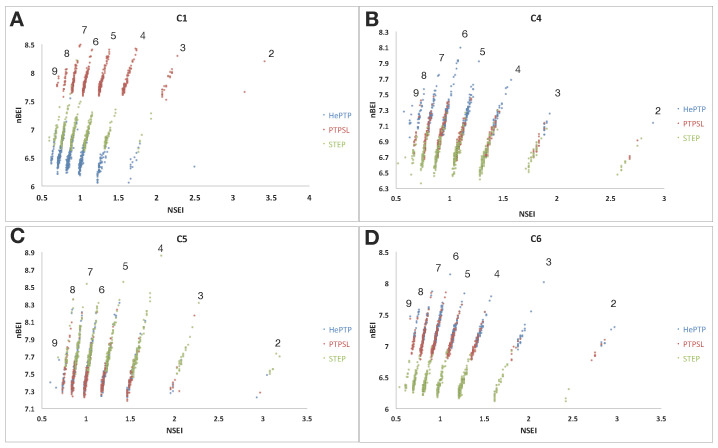
VSpipe NSEI/nBEI plot analysis of selected ligands (top 500 ranked by ∆G) for clusters 1, 4, 5 and 6 for the KIM-PTPs (HePTP, PTPSL and STEP). Each panel is an NSEI/nBEI plot of the cluster ligands for the three KIM-PTPs: (**A**) cluster 1 binders, (**B**) cluster 4 binders, (**C**) cluster 5 binders, and (**D**) cluster 6 binders. The dots represent individual ligands and the colouring is as follows: HePTP (blue), PTP-SL (red), and STEP (green). Each diagonal line of compounds represents a specific NPOL (number of N and O atoms in the compound) as given by the number above each diagonal line.

The highest average binding score for HePTP and STEP was found in the C5 cluster with an average ΔG of −8.2 and −8.5 kcal/mol, respectively. In contrast, the highest average score in PTP-SL was for the C1 cluster with ΔG of −8.7 kcal/mol (Table 2). Although the ΔG values may offer an estimate of the binding affinity, they provide little information on the drug-like nature of the ligands. This can be assessed using the ligand efficiency index (LEI) plots generated by VSpipe [29] (Figure 2). These plots correlate the binding efficiency of the ligands with two important physiochemical properties that impact on the pharmacokinetic and oral bioavailability of the compounds: molecular weight and polar surface area. Optimisation of these properties, together with potency, aid the development of drug-like compounds [31]. The SEI and BEI efficiency indices are of particular interest where SEI is the surface-binding efficiency index in respect to polar surface area, and BEI is the binding efficiency index related to the molecular weight. The equations for these LEIs are provided in the methods.

The application of these plots to PTP drug discovery has been demonstrated in the literature [32,39], and shown to unveil unique opportunities to explore alternative functional sites to the highly conserved active site [29,30]. The NSEI/nBEI plots generated by VSpipe for each targeted docking were used to assess how the LEI profiles of clusters 1, 4, 5 and 6 vary across the KIM-PTPs (Figure 2). In these plots the ligands are sorted according to the number of polar atoms (nitrogen, oxygen) or NPOL planes, along which efficiency increases from the bottom to the top of each [39] (Figure 2).

For C1, the distribution of binders was similar for all three KIM-PTPs, with the larger number of ligands along the NPOL 5 to 7. The best scorer was located on NPOL 7 for all three KIM-PTPs, demonstrating a high dependency of efficient binders at this site (active site) on polarity. The most efficient ligands at any NPOL line were binders of PTP-SL, whilst the least efficient on all NPOL lines were binders of HePTP (Figure 2A). This is in good agreement with the ΔG average values from the VSpipe docking (Table 2).

Analysis of the C4 binders (Figure 2B) also showed a similar distribution of binders across NPOL lines for all three KIM-PTPs, with the highest populations on the NPOL 5 and 6 lines. Differences were observed for the best scorer, located on NPOL 6 for HePTP and STEP, and NPOL 8 for PTP-SL, indicating that PTP-SL binders show higher polarity. The most efficient binders across all NPOL planes are HePTP ligands, whilst the C4 binders of STEP are the least efficient.

C5 binders (Figure 2C) show the highest distribution on the NPOL 4 to 6 lines, with the best scorers located on NPOL 5 for HePTP and PTP-SL, and on NPOL 4 for STEP. Thus, the site associated with C5 favours more hydrophobic binders than the other clusters, particularly for STEP. The most efficient ligands on each NPOL line were binders of STEP, and the least efficient binders were for PTP-SL. STEP C5 binders reside in the best chemical biological space (top-right quadrant) in contrast with its binders at the other clusters (bottom-left), thus this site represents a good opportunity to exploit selectivity for this phosphatase over the others in the same subfamily.

For C6 (Figure 2D), the distribution of binders was also similar for all three KIM-PTPs, with the highest distribution on the NPOL 5 to 7 lines, and the best scorers located on NPOL 6. This suggests there is little difference in the preference of polarity across the KIM-PTPs at the site associated with cluster 6. The most efficient ligands on each NPOL line were binders of HePTP and PTP-SL binders, whilst the least efficient were binders of STEP.

In summary, the LEI analysis, together with the druggability scores, support the notion that there are sufficient differences between the three KIM-PTPs that could be exploited to develop selectivity using different binding sites. For example, C1 for PTP-SL, C4 for HePTP or C5 for STEP, as discussed above.

### 2.3. Structural Analysis of the Cluster Binding Sites

The analysis of the chemical–biological space distribution for the cluster ligands highlighted differences between the three KIM-PTPs. To understand these differences and the determinants for ligand affinity and polarity, we analysed the structural properties of the corresponding binding sites.

C1 ligands, binding at the active site, showed a clear preference for PTP-SL with a ΔG average of −8.7 with respect to HePTP (−6.9) or STEP (−7.4) (Table 1). The PTP-SL active site also has a higher druggablity score than the active site of the other KIM-PTPs (Table 2). The main reason for these differences is, in part, the volume of the pocket that for PTP-SL is 428 Å^3^, whereas for STEP is only 280 Å^3^. The percentage of polar residues at this site was also lower for PTP-SL (45%) than for STEP (53%), and consequently cluster binders reside in a more drug-like space. For HePTP, the pocket is larger because it merges with the open pocket, making comparisons difficult.

**Figure 3:**
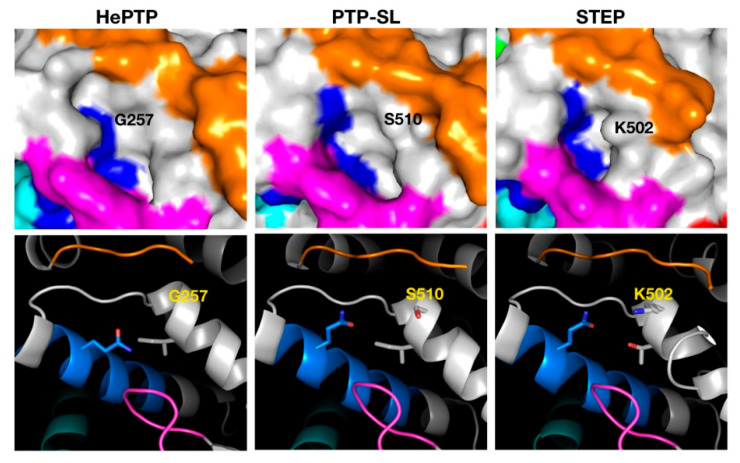
Structural analysis of the pocket associated with cluster 4 across the three KIM-PTPs (HePTP, PTPSL and STEP). In the top panels the three KIM-PTPs are represented as surfaces, whilst in the bottom panels they are shown in cartoon format, with key residues shown as sticks and labelled accordingly. In both panels the colouring is as follows: KIM-PTP specific motifs K3 (orange), K5 (magenta), K6 (cyan), and K7 (blue).

The binders of the highest affinity in C4 were those that targeted HePTP, whilst STEP C4 binders had the lowest affinity and resided in the least drug-like chemical–biological space. The associated pocket of C4 in HePTP also had the highest drugability score (Table 2). The main differences in the C4 pocket across the KIM-PTPs are its size and the access to this pocket. In HePTP, the C4 pocket is the largest with 324 Å^3^. However, in STEP, access to the pocket is partially blocked by the side chain of K502, resulting in a considerably smaller pocket at 187 Å^3^. In HePTP, this residue is a glycine, G257, and in PTP-SL a serine, S510 (Figure 3), both having smaller size side chains than K502. The C4 pocket for STEP is also more polar (composed of 67% polar residues) than that of the pocket for HePTP (39% polar residues) and PTP-SL (50% polar residues). This is, in part, a consequence of the replacement of an isoleucine in PTP-SL and HePTP with a threonine in STEP. This size difference and access to the pocket would be less favourable during docking, thus explaining why a low-density cluster is observed for STEP.

**Figure 4:**
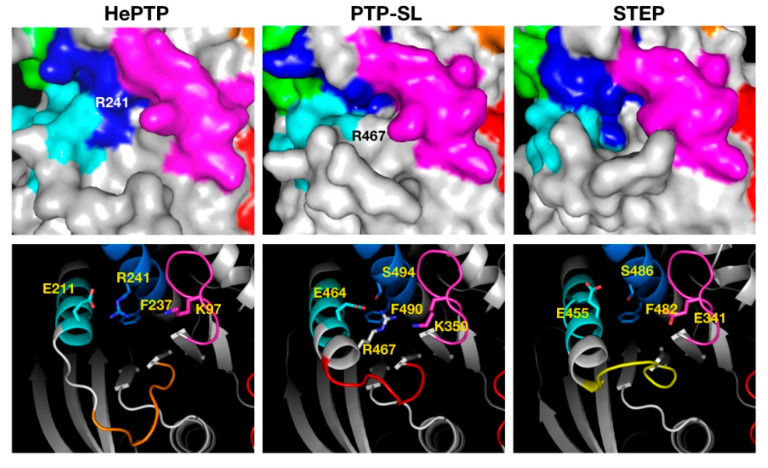
Structural analysis of the pocket associated with cluster 5 across the three KIM-PTPs (HePTP, PTPSL and STEP). In the top panels the three KIM-PTPs are represented as surfaces, whilst in the bottom panels they are shown in cartoon format, with key residues shown as sticks and labelled accordingly. In both panels the colouring is as follows: KIM-PTP specific motifs K5 (magenta), K6 (cyan), and K7 (blue). In the bottom panel the difference in the conformation of the loop that forms the lip at the bottom entrance of the pocket is also coloured as follows: HePTP (orange), PTPSL (red), and STEP (yellow) to aid visualisation.

The C5 cluster (Figure 4), binding across K5, K6 and K7, was the only example where binders showed higher affinity for STEP and ligands resided in the most drug-like space. Inspection of the C5 binding pocket for the three KIM-PTPs shows a deeper pocket for STEP that may afford higher binding affinity. A key residue at the bottom of the pocket is the phenylalanine residue of the K7 motif (Figure 4), a conserved residue across the KIM-PTPs that adopts the same conformation in all three proteins. The reason why PTP-SL ligands appear to have lower affinity is the presence of R467 that blocks access to F490 and results in a smaller opening of the pocket (Figure 4). This residue is an alanine in STEP, and in HePTP this region is a disordered loop instead of helical, thus leaving it more open to access the pocket (Figure 4). However, in HePTP, R241 at the top of the pocket partially obstructs access to F237. This residue is a serine in both PTP-SL and STEP (Figure 4).

Another important difference between the three KIM-PTPs is the loop that forms a lip at the bottom entrance of the pocket (coloured yellow, red and orange for STEP, PTP-SL and HePTP, respectively, in Figure 4). The sequence of this eight-residue loop (ten residues in HePTP) is not conserved, and this is reflected on its conformation in the crystal structures. In HePTP, it adopts a wide-open conformation, leaving a larger pocket. In STEP, it adopts a more closed position, creating a deep pocket, and in PTP-SL it adopts an intermediate position. The binding site associated with C5 is the only cluster that may deliver selectivity for STEP over the other two KIM-PTPs, given the considerable differences in pocket architecture and sequence. This pocket also shows the highest druggability scores for all KIM-PTPs and the lowest percentage of polar residues.

**Figure 5:**
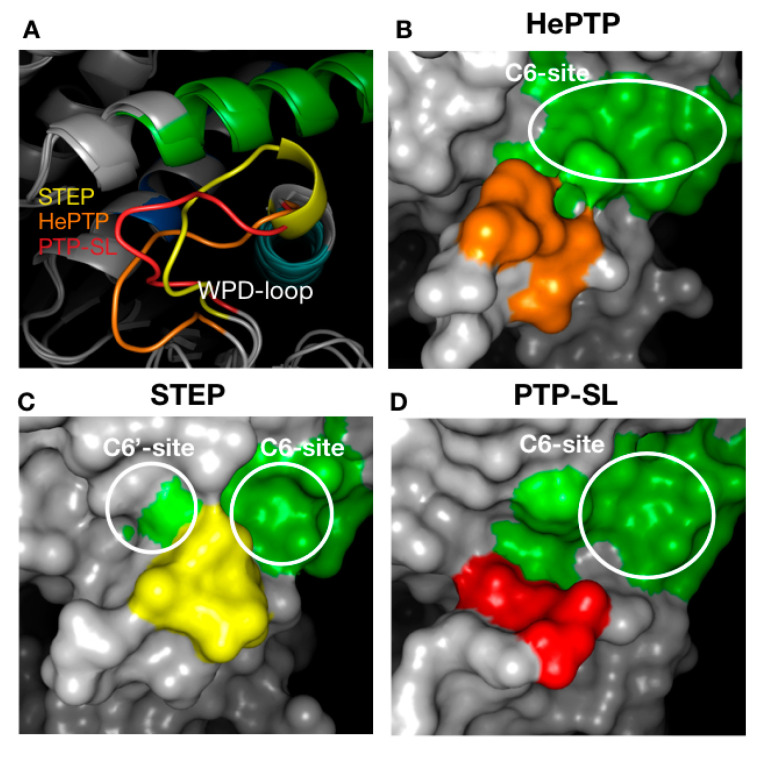
Structural analysis of the pocket associated with cluster 6 across the three KIM-PTPs (HePTP, PTPSL and STEP). (**A**) Different conformations of the WPD loop across the KIM-PTPs are shown in cartoon format. (**B**–**D**) The effect of the position of WPD-loop on the size and shape of the pocket associated with cluster 6 is shown, with the three KIM-PTPs being represented as surfaces. The colouring is as follows: the K8 motif is green, whilst the WPD loop is coloured orange (HePTP), yellow (STEP), and red (PTP-SL).

Finally, the highest affinity binders in C6 were those that targeted HePTP, whilst STEP binders had the lowest affinity and resided in the least drug-like chemical–biological space. The differences in surface topography at the C6 binding site can be explained when considering the conformation of the WPD-loop and its position in respect to the helix that precedes the K3 motif (Figure 5A). In the case of HePTP and PTP-SL, the WPD-loop is far away from the helix (>9 Å), thus generating space for a larger pocket (Figure 5B,D). For STEP, the loop is considerably closer to the helix (<7 Å), thus splitting the pocket into two smaller pockets C6 and C6′ (Figure 5C).

Furthermore, the C6 and C6′ sites in STEP contained a much higher percentage of polar residues, thus ligands there reside in the least favoured chemical–biological space (bottom-left quadrant of the NSEI/nBEI plot) (Figure 2D). Exploiting this pocket for inhibitor development may afford selectivity to HePTP and PTP-SL over STEP. Further to this, selectivity may be possible between HePTP and PTP-SL given the differences in size and shape of the C6-site between both proteins. However, all pockets associated with C6 have poor druggability scores relative to the pockets associated with C4 and C5 (Table 2).

### 2.4. Blind Docking Clusters Are Subfamily Specific

**Figure 6:**
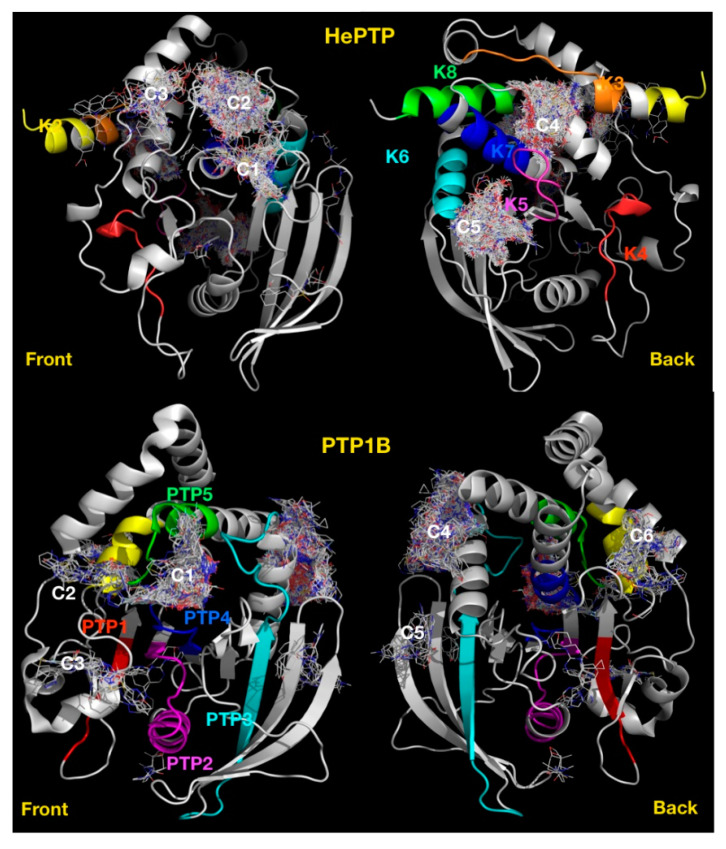
Blind docking cluster comparison between HePTP and PTP1B. The structure of HePTP (**top**) is shown as a grey cartoon, key functional KIM specific motifs [33] have been coloured on the HePTP structure, K2 in yellow, K3 in orange, K4 in red, K5 in magenta, K6 in cyan, K7 in blue, K8 in green. Compounds are shown as lines. Ligand clusters with more than ten compounds are numbered: C1, active site; C2, open form pocket; C3, substrate-binding site; C4, centred on K3, K5 and K7 motifs; C5, centred on K5–K7 motifs. The structure of PTP1B is shown as a grey cartoon (**bottom**), front view and back view. Clusters are numbered: C1, active site; C2, secondary pTyr site; C3, centred on L41; C4, allosteric binding site; C5, centred on V155; and C6, centred on E252. Key PTP motifs [33] have been coloured on the structure: PTP1 in red; PTP 2, magenta; PTP 3, cyan; PTP 4, blue; PTP 5, green; and PTP6, yellow.

Most of the clusters identified for the KIM-PTPs are centred on motifs that are specific to this subfamily of PTPs [33]. To test the use of blind docking as a PTP profiling approach based on cluster distribution, we carried out blind docking against PTP1B in its open form (PDB ID: 1T4J) with the Asinex fragment library. This resulted in the identification of six clusters of fragments (Figure 6), together with four low-density binding sites (less than ten fragments at each site). The clusters located at the following motifs or regions: C1, the active site [34]; C2, the secondary phospho-tyrosine binding site [40]; C3, a site centred on L41; C4, an allosteric binding site [25]; C5, a site centred on V155; and C6, a site centred on E252.

The only common cluster between KIM-PTPs, exemplified by HePTP and PTP1B, is the cluster at the active site (C1), with all other HePTP clusters locating at KIM-PTP specific motifs. Thus, binders at these sites may deliver KIM-PTP specific inhibitors over the classical PTP1B.

### 2.5. Functional Correlation with Cluster Distribution

**Figure 7:**
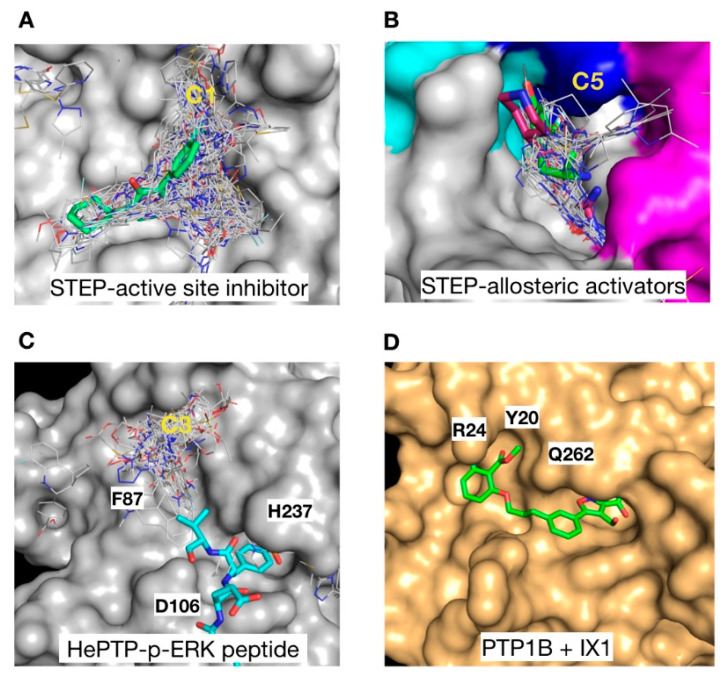
Functional correlation with cluster distribution. (**A**) Surface representation of STEP with C1 cluster (grey lines) overlaid onto a STEP active site inhibitor (green sticks) [18]. (**B**) STEP C5 cluster (grey lines) overlaid onto a STEP allosteric activator (magenta sticks) [19]. The STEP surface is coloured as follows: K5 in magenta, K6 in cyan, and K7 in blue. (**C**) Structure of HePTP with C3 cluster (grey lines) shown together with the HePTP substrate p-ERK (cyan sticks) [37]. (**D**) Structure of PTP1B as a gold surface in complex with the bi-dentate inhibitor IX1 (green sticks) [41]. Key residues in the secondary p-Tyr binding site are labelled.

In an effort to validate the functional importance of the cluster distribution, we mapped the position of the identified clusters to that of reported substrates, inhibitors and activators of KIM-PTPs. In the case of STEP, the C1 cluster overlaps with the core structure of a STEP active site inhibitor (5OW1 [18]), and it extends towards a sub-pocket nearby suggesting a possible target for expansion in future development of active site inhibitors (Figure 7A). Blind docking also identified the site of the recently developed allosteric activators of STEP (6H8S, 6H8R, [19]). The C5 cluster overlaps with these allosteric activators, and highlights potential regions for additional interactions at a secondary pocket where ligands bind (Figure 7B).

The binding site associated with C3 in HePTP (Figure 7C) is particularly interesting since a cluster at this site was not observed for either of the other two KIM-PTPs or PTP1B, suggesting it may be a key driver of selectivity. The C3 cluster partially overlaps with the position of the p-ERK peptide substrates binding in this region (3D42 [37]), and many of the ligands form interactions with H237 and the mutated D106, both critical residues in regulating substrate binding. This may open up avenues for the development of bi-dentate or pseudo-peptide inhibitors that mimic the biological substrates of HePTP. Bi-dentate inhibitors that target the C3 binding region may afford selectivity over PTP1B, where the position of Y20, R24 and Q262 in the pTyr secondary site nearby would clash with the C3 ligands. Conversely, double-site inhibitors of PTP1B (1XBO [41]) exploit the pTyr secondary site (Figure 7D), which in HePTP is blocked by F87, a conserved residue in all KIM-PTPs.

## 3. Materials and Methods

### 3.1. Preparation of the Models

The catalytic domain sequence of HePTP was downloaded from www.uniprot.org, accessed on 2 November 2020. The 3D structure used as template for the open form of HePTP was obtained from the Protein Data Bank (https://www.rcsb.org, accessed on 2 November 2020). For the modelling, the basic option in Modeller was utilised, with PDB ID: 3O4U. The residue sequences ^257^DHQTP^261^ and ^196^QLREGKEKC^204^ were modelled. Residues ^256^P and ^262^E flanking ^257^DHQTP^261^ were kept rigid. Five complete models for both regions were generated and the model for each loop with the lowest modeller objective function score was selected for subsequent docking.

The structures of PTP-SL (PDB ID: 1JLN), STEP (PDB ID: 2BV5) and PTP1B (PDB ID: 1T4J) were used as starting models for docking without further modifications.

### 3.2. Virtual Screening with VSpipe

VSpipe v1.0 [29] was used for all the blind and targeted docking, as well as the generation of the ligand efficiency (LEI) plots. VSpipe [29] is a semi-automated pipeline that uses MGLTools, AutoDock tools [42], OpenBabel [43], and in-house Python and R scripts to perform structure-based virtual screening.

Blind docking was performed with the VSpipe-Vina [29] option for all targets using a grid spacing of 0.375 Å, and a box size sufficient to include the whole protein structure. Blind docking was carried out with the Asinex BB v123 SD library (www.asinex.com, accessed on 15 April 2018) that contains 6243 chemical fragments. The compounds are low molecular weight building blocks and abide by the following rules molecular weight 120–250, cLogP < 2.5, HBA < 7, HBD < 4.

For the analysis of HePTP, we used the following grid centre: x = 12.254, y = 17.806, z = −22.012. For PTP-SL, we used: x = 18.520, y = −5.430, z = 8.985. For STEP, we used: x = 11.136, y = −10.835, z = −16.125. For the analysis of PTP1B, we used the following grid centre: x = 56.500, y = 31.333, z = 22.113.

Targeted docking was performed with VSpipe-Autodock [29] using the default standard protocol for non-covalent ligands. The box size for all proteins and sites explored was 15 × 15 × 15 Å^3^, and a spacing of 0.375 Å was used. Docking was done with the Chembridge Diverset library composed of 50,000 compounds. This library contains a diverse collection of lead-like molecules (www.cambridgemedchemconsulting.com/DDResources/Hit_iden/frag_collection.html, accessed on 15 April 2018).

The parameters used for each protein are as follows. For HePTP targeted docking, the grid centre used for the cluster 1 was: x = −7.367, y = 20.032, z = −17.759; for cluster 3, it was: x = −28.392, y = 21.340, z = −17.759; cluster 5 was: x = −5.493, y = 0.912, z = −12.290; for cluster 6, it was: x = −27.866, y = 1.499, z = −6.069; for cluster 7, it was: x = −32.148, y = 5.340, z = −23.870. For PTPSL, the grid centre used for the cluster 1 was: x = 14.125, y = −8.987, z = 6.586; for cluster 3, it was: x = 35.369, y = −16.222, z = 3.589; for cluster 6, it was: x = 40.107, y = −10.355, z = 24.945; for cluster 7, it was: x = 31.332, y = 8.029, z = 18.912. For STEP, the grid centre used for the cluster 1 was: x = 7.321, y = −18.826, z = −17.129; for cluster 3, it was: x = 19.086, y = −18.085, z = −38.811; for cluster 6, it was: x = 31.193, y = −5.244, z = −21.540; for cluster 7, it was: x = 25.555, y = 6.644, z = −15.12.

All computational tasks were carried out at the Computational Shared Facility (CSF) at the University of Manchester.

### 3.3. Ligand Efficiency (LEI) Plots

All LEI plots used to analyse the binding clusters in this study were generated from VSpipe using the following equations taken from [32]:

NSEI (−log_10_K_i_/NPOL) vs. nBEI (−log_10_[(K_i_/NHEA)]), and SEI (p(K_i_)/(PSA/100 Å^2^) vs. BEI (p(K_i_)/*M*_W_(kDa)). The plots help to visualise the chemical–biological space for each ligand cluster obtained from the targeted docking.

### 3.4. Pocket Druggability Predictions

Pocket druggability predictions were performed using the DoGSiteScorer server https://proteins.plus/#dogsite, accessed on 27 May 2020. The software makes predictions regarding protein pockets using a variety of physiochemical and geometric discriminators to yield a mean druggability score; the greater the score, the more druggable the pocket [38].

Structural analyses and figures were prepared with MacPyMOL: PyMOL v1.8.0.3 Enhanced for Mac OS X (Schrödinger LLC, New York, NY, USA).

## 4. Conclusions

In this work we present the use of blind docking with the virtual screening tool VSpipe, to identify allosteric ligand binding sites that are specific to the subfamily of KIM-PTPs. The VSpipe blind docking on KIM-PTPs (HePTP, STEP, PTP-SL) resulted in a unique cluster distribution, different to that observed previously for the classic PTP1B [29]. Therefore, the VSpipe blind docking approach may offer a way of profiling PTPs at the subfamily level.

Differences in the binding site druggability and the profile of the ligand clusters between each KIM-PTP highlighted the potential for selective inhibition, even within the subfamily. Importantly, several of the clusters identified reside at sites of known functional relevance for activity or substrate binding specific to the KIM-PTPs. An assessment of the drug-like properties of ligands at each binding site was facilitated by analysis of LEI plots generated by VSpipe targeted docking at the cluster sites. From this, it was ascertained that several of the KIM-PTP specific binding sites selected ligands residing in a more drug-like chemical space than those binding to the active site, which is highly polar. Differences were observed when comparing the ligand clusters from different KIM-PTPs, either in respect to their position or their LEI profile.

Structural analysis of the most druggable sites allowed a rationalisation of the differences in binding and in the LEI profiles across the KIM-PTP subfamily of enzymes, providing a rationale for further inhibitor development to achieve selectivity.

VSpipe provides a quick, easy and cost-effective stepwise approach for the identification of new sites to target for drug development, particularly allosteric sites. The combined analysis of the targeted docking with that of the LEI profiles of ligand clusters facilitates the identification of specific binding sites that may afford selective inhibition and development of drug-like binders. Ultimately, this work opens up a new paradigm for the development of KIM-PTP inhibitors, as well as phosphatase inhibitors, and more widely to any drug target of interest.

## Figures and Tables

**Table 1:** Druggability scores calculated by DoGSiteScorer [38] of the pockets associated with clusters 1, 4, 5 and 6 for HePTP, PTPSL and STEP.

PTP	Pocket (Site of Cluster)	Druggability Probability	Vol. Hull/Å3	% Polar Residues
HePTP	C1/C2	0.83	617	39
HePTP	C4	0.61	324	39
HePTP	C5	0.81	522	29
HePTP	C6	0.27	135	10
PTP-SL	C1	0.61	428	45
PTP-SL	C4	0.55	259	50
PTP-SL	C5	0.74	604	29
PTP-SL	C6	0.37	177	20
STEP	C1	0.51	280	53
STEP	C4	0.24	187	67
STEP	C5	0.72	475	37
STEP	C6	0.34	155	50
STEP	C6′	0.23	134	50

**Table 2:** Binding affinities (ΔG) of the top 500 binders from targeted docking of the Chembridge Diverset library at the pockets associated with clusters 1, 4, 5 and 6 for HePTP, PTPSL and STEP.

PTP	Cluster	ΔG Low (kcal/mol)	ΔG High (kcal/mol)	ΔG Average (kcal/mol)
HePTP	1	−6.7	−8.3	−6.9
HePTP	4	−7.7	−9.0	−7.9
HePTP	5	−8.0	−9.4	−8.2
HePTP	6	−7.7	−9.1	−7.9
PTP-SL	1	−8.5	−9.8	−8.7
PTP-SL	4	−7.3	−8.2	−7.5
PTP-SL	5	−8.0	−9.3	−8.2
PTP-SL	6	−7.4	−8.7	−7.6
STEP	1	−7.2	−9.1	−7.4
STEP	4	−7.0	−8.0	−7.2
STEP	5	−8.3	−10.1	−8.5
STEP	6	−6.6	−7.5	−6.8

## Data Availability

The Linux version of VSpipe-local mode and documentation are available at https://github.com/sabifo4/VSpipe.

## Supplementary Materials

The following are available online at https://www.mdpi.com/article/10.3390/ijms222212206/s1.
