# Supplementary material for: A New Paradigm for KIM-PTP Drug Discovery: Identification of Allosteric Sites with Potential for Selective Inhibition Using Virtual Screening and LEI Analysis
Source: Int J Mol Sci. 2021 Nov 11;22(22):12206. doi: 10.3390/ijms222212206 (PMC8624330; doi:10.3390/ijms222212206)
Supplement: Supplementary file 1 [file ijms-22-12206-s001.zip › ijms-1439395-supplementary.pdf]

Supplementary Material

A New Paradigm for KIM-PTP Drug Discovery: Identification of Allosteric Sites with Potential for Selective Inhibition Using Virtual Screening and LEI Analysis

James Adams [1], Benjamin P. Thornton [1] and Lydia Tabernero [1,2,3,*]

[1] School of Biological Sciences, Faculty of Biology Medicine and Health, Manchester Academic Health Science Centre, University of Manchester, Manchester M13 9PT, UK; jadams_91@hotmail.co.uk (J.A.); benjamin.thornton-3@postgrad.manchester.ac.uk (B.P.T.)

[2] Lydia Becker Institute for Immunology and Inflammation, University of Manchester, Manchester M13 9PT, UK

[3] Antimicrobial Resistance Network, University of Manchester, Manchester M13 9PT, UK

* Correspondence: Lydia.Tabernero@manchester.ac.uk; Tel.: +44-(0)-161-275-7794

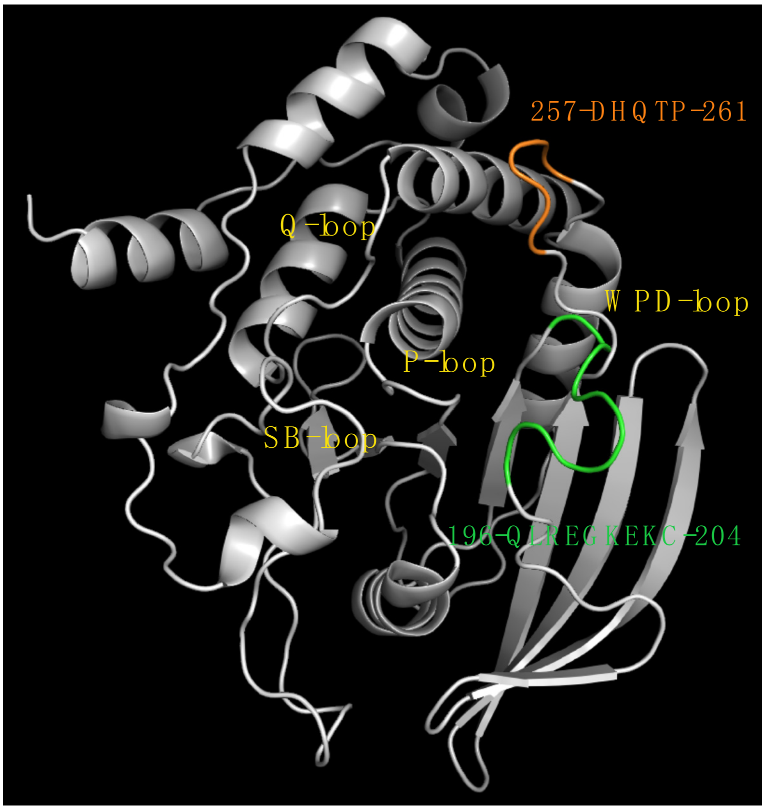

Supplementary Figure S1. Complete models generated using modeller for the open forms of HePTP. Model for the open form (PDB ID: 3O4U) shown as grey cartoon with key loops labelled (yellow). The loops that were modelled are coloured as follows, ^{196}QLREGKEKC^{204} is shown in green, and ^{257}DHQTP^{261} is shown in orange.
